# Clinical implications for dopaminergic and functional neuroimage research in cognitive symptoms of Parkinson’s disease

**DOI:** 10.1186/s10020-021-00301-7

**Published:** 2021-04-15

**Authors:** Shigeki Hirano

**Affiliations:** Department of Neurology, Graduate School of Medicine, Chiba University, Chiba, Japan

**Keywords:** Parkinson’s disease, Perfusion, Glucose metabolism, Dopamine, Dual syndrome hypothesis, Caudate nucleus, Dementia, Cognitive impairment

## Abstract

Evidence from dopaminergic image and cerebral blood flow/metabolism images have shed light on symptomatology of cognitive aspects in brain physiology of healthy human as well as patients with Parkinson’s disease. Cognitive impairment in Parkinson’s disease is characterized by executive, visuospatial, attentional disturbances. Dopaminergic system includes triadic parallel pathways. The mesostriatal pathway consist of posterolateral putamen and motor areas, the mesocortical pathway of dorsal caudate nucleus and dorsolateral prefrontal cortex, and the mesolimbic pathway of ventral striatum, anterior cingulate cortex. The mesocortical pathway is responsible for the executive function which may change by administration of dopaminergic medication. The mesolimbic pathway is associated with motivation and reward prediction which may result in depression or apathy when dopamine level was suboptimal, impulse control disorder and punding when dopamine was over the optimal level. Abnormal brain metabolism/perfusion related to cognitive impairment in Parkinson’s disease are relatively reduced activity located in frontal and parietal association areas and relatively increased activity in the cerebellum. In the anterior brain, the mesocortical pathway, is responsible for verbal memory and executive function, which originates with caudate dopaminergic system and account for mild cognitive impairment of Parkinson’s disease. The posterior brain system which includes the parietal, temporal, and occipital cortices, is responsible for the memory and visuospatial function, and related to cholinergic dysfunction and possibly glucocerebrosidase gene variants, relating to dementia in Parkinson’s disease. The role of cerebellum in Parkinson’s disease remains unclear but emerging evidence suggests that it may relate to the sequencing detection and affective symptoms. The dual syndrome hypothesis is helpful for understanding the mechanism of cognitive impairment in Parkinson’s disease and optimal symptom management.

## Introduction

Neuroimaging results, notably dopaminergic image and cerebral blood flow/metabolism images, have contributed evidence to interpret the symptomatology of cognitive aspects of human brain physiology, including in patients with Parkinson’s disease (PD). PD is a movement disorder with pathological hallmarks of progressive neurodegeneration of nigrostriatal dopaminergic neurons and the presence of Lewy bodies in the surviving neurons. The physiological role of the dopaminergic system in humans has been elucidated using PD as a model of selective dopaminergic cell loss. However, it has been recently recognized that in PD, multiple neural systems are variably involved, such as the serotonergic, noradrenergic, and cholinergic systems, which may, in part, explain the non-motor symptoms of PD, including cognitive, psychiatric, autonomic, sleep, and sensory symptoms. Of note, by cross-sectional observations, mild cognitive impairment was observed in 40% of patients with PD and dementia develops in approximately 30% of PD patients, which may further increase by longitudinal observation, and has a significant impact to the lives of PD and their caregivers (Aarsland et al. 1996; Williams-Gray et al. 2007a; Baiano et al. 2020). Symptomatic and prognostic heterogeneity is a major challenge for understanding dementia in PD. Therefore, capturing the clinical symptoms and objective assessments are crucial for adequate management of PD patients. In PD-related cognitive impairment, neuropsychological tests as well as neuroimaging plays an important role for the clinical as well as research settings. A number of underlying pathophysiologies account for the cognitive impairment of PD; (i) abnormal reduction of neurotransmitters (dopamine, acetylcholine, noradrenaline, serotonin); (ii) the presence of cortical Lewy bodies; (iii) concomitant Alzheimer’s disease (AD) pathology (i.e. hippocampal atrophy and abnormal disposition of beta amyloid and phosphorylated tau); (iv) concomitant vascular pathology; and (v) pharmaceutical interaction in cognition. In addition, the normal aging process complicates the issue, since most of the PD subjects develop later in the life.

The current review covers the brain dysfunction of cognitive aspects in PD demonstrated by neuroimaging findings. First, the characteristics and the risk of cognitive impairment of PD are discussed. Second, dopaminergic systems of caudate nucleus and ventral striatum are reviewed with regard to the cognitive aspects. Third, the function of brain regions related to cognition, shown by glucose metabolism, cerebral perfusion imaging or functional magnetic resonance image (fMRI), are discussed. Lastly, evidence has integrated these systems to introduce an idea of ‘dual syndrome hypothesis’. This review will focus on mild cognitive impairment (MCI) and dementia in PD, while dementia with Lewy bodies is left for other references.

## Clinical aspects of cognitive impairment in PD

Cognitive impairment of PD is characterized by the impairment of executive function (decision-making, planning, problem solving, maintaining or shifting attention, and inhibition of habitual responses), visuospatial performance, attention, bradyphrenia (psychomotor slowing), mild to moderate memory disturbance (mainly retrieval), and internally cued behavior. Executive function can be assessed representatively by Wisconsin Card Sorting Test, Tower of London task, or verbal fluency, which mirrors frontal lobe function. Early stage PD may exhibit cognitive impairment, characterized by impairments in executive function and cognitive flexibility, including reversal learning, response inhibition, planning, attentional set shifting, action selection, and risk-reward-related decision, and leads to inability to adapt behavior to a novel or changing environment (Rowe et al. 2019). In the medication OFF state, PD patients are impaired at planning or at switching from one task to another, while showing less impairment on risk-taking paradigms or probabilistic reversal learning (Cools et al. 2019). Cross-sectional data-driven analysis of cognitive impairment in PD demonstrated that one patient group had mild bradyphrenia with executive dysfunction, and lower verbal episodic memory, another patient group had mild but generalized decrement with notable decline in visuospatial function and another patient group was characterized by severe bradyphrenia and memory disturbances. The remaining group was either cognitively normal or severely impaired (Dujardin 2013).

Risk factors for developing dementia in PD include age, severe motor disability, male sex, visual hallucination, postural instability and gait disturbance type, bradykinesia relative to tremor, and poor response to levodopa (Williams-Gray et al. 2007a; Caparros-Lefebvre et al. 1995; Burn and McKeith 2003; Irwin et al. 2013). In contrast, executive dysfunction, such as phonemic fluency, was independent from these abovementioned risks, with a better prognosis, and associates with catechol-O-methyl transferase (COMT) variant, indicates its relation to dopaminergic function (Williams-Gray 2009). COMT is the main regulatory factor in the frontal cortex, since the dopamine transporter (DAT) is relatively spared in this brain region. A single methionine (Met) to valine (Val) substitution at 158 induces an up to four-fold difference in enzymatic efficacy. In other words, dopamine metabolism may differ between individuals, depending on the *COMT* variants. Val carriers have higher COMT activity, resulting in lower dopamine in prefrontal regions, while those having Met have decreased COMT activity and higher prefrontal dopamine levels. Glucocerebrosidase (GBA) gene variant carriers in PD have worse cognition, motor symptoms, and shorter survival than those who does not have the variant, implying GBA as a risk gene for developing dementia (Aarsland 2017). In sum, there are subtypes of cognitive impairment in PD and the contributing risk factors are, pharmacological status, the disease severity, and the gene variants, including *COMT* and *GBA*.

## Dopaminergic neuroimage in cognitive impairment of PD

### Caudate nucleus

**Fig. 1:**
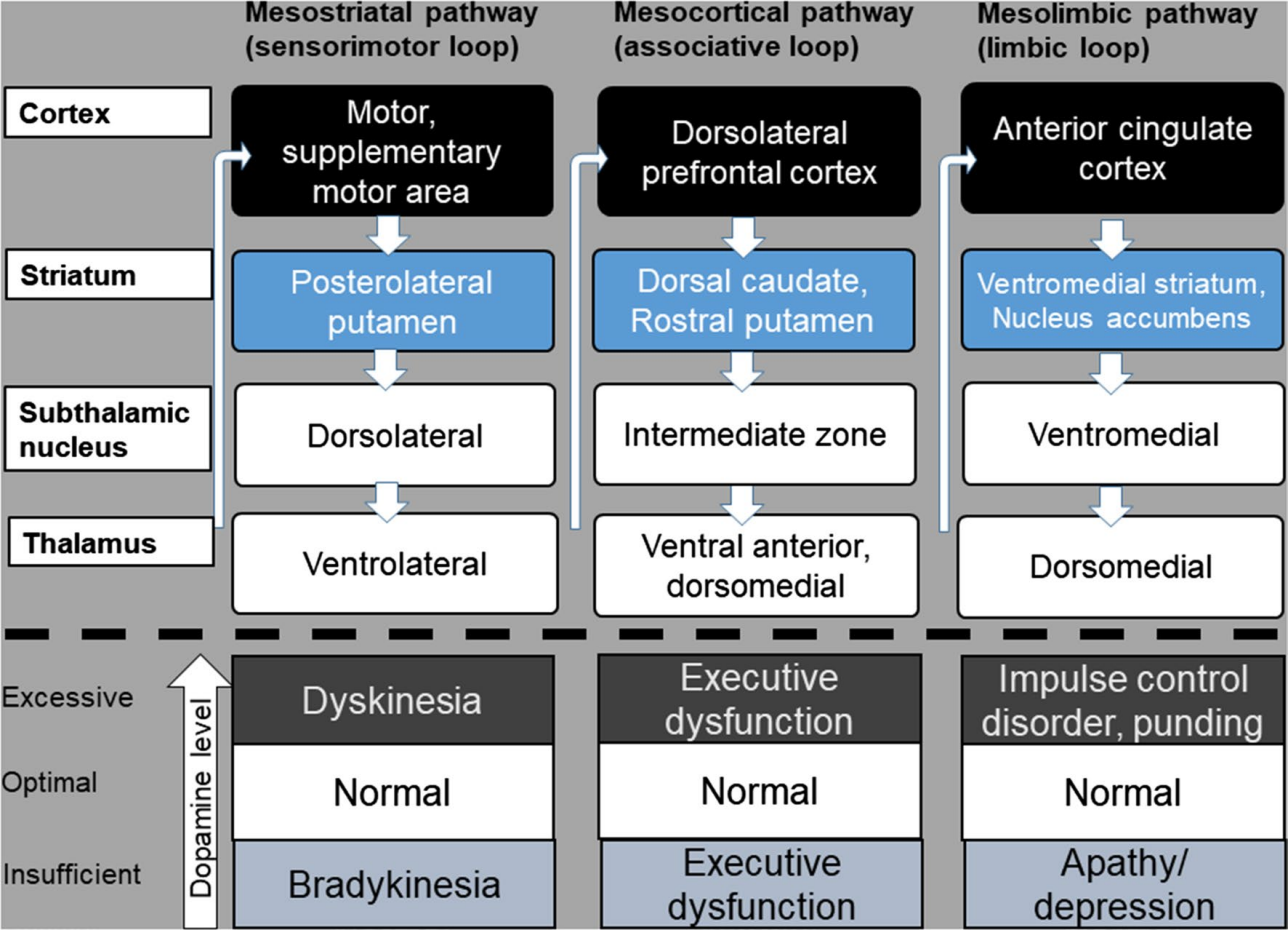
Triadic parallel cortico-striatal-pallido-thalamo-cortical loop and the clinical relevance in relation to the striatal dopamine level (modified from Rowe et al. (2019) and Volkmann et al. (2010))

A triadic subdivision of the parallel striatal projections exists in the dopaminergic system, the mesostriatal, mesocortical, and mesolimbic pathways. These consist of parallel cortico-striatal-pallido-thalamo-cortical (CSPTC) loops (Fig. 1) (Rowe et al. 2019; Alexander et al. 1986; Cummings 1993; Volkmann et al. 2010). The mesostriatal dopaminergic circuit is comprised of ventral midbrain, posterolateral putamen, dorsolateral subthalamic nucleus (STN), and primary motor cortex, and responsible for motor dysfunction. The mesocortical dopaminergic circuit is comprised of the head of the caudate nucleus, rostral putamen, intermediate zone of STN, and the dorsolateral prefrontal cortex. The mesolimbic dopaminergic circuit is comprised of the nucleus accumbens, ventromedial striatum, rostral ventral, ventromedial STN, anterior cingulate cortex. These circuits were also demonstrated by a resting state fMRI study in PD, showing robust connectivity between the posterior putamen and cortical motor areas, the anterior putamen and the pre-supplementary motor area and anterior cingulate cortex, the caudate nucleus and dorsal prefrontal cortex (Helmich 2010). In PD, the mesostriatal dopaminergic circuit is most prominently affected, whereas in the early stage of the disease, the mesocortical and mesolimbic dopaminergic circuits are relatively preserved (Rinne 2000; Ma 2002). Preservation or dysfunction of these circuits explains the relationship between symptoms and dopaminergic state; the mesostriatal system is responsible for bradykinesia in the hypodopaminergic, and dyskinesia in the hyperdopaminergic state; the mesocortical system is responsible for executive function which declines in the hypo- and hyperdopaminergic state; and the mesolimbic system is responsible for apathy/depression in hypo-dopaminergic state, impulse control disorder or punding in the hyperdopaminergic state (Fig. 1).

In healthy subjects, the nigrostriatal dopaminergic system steadily declines by normal aging at a rate of 3 to 7% per decade (Kaasinen et al. 2015; Matsuda 2018). This age-related decline of dopaminergic system may correspond to aspects of ‘fluid intelligence’ (processing speed, problems solving ability) which decline by aging (Ryan et al. 2000). To further understand the physiological role of the dopaminergic system and cognitive function, the association between dopaminergic imaging and cognition in healthy subjects have been studied. Thirty healthy subjects were examined by ^123^I-FP-CIT single photon emission computed tomography (SPECT) and Wechsler Adult Intelligence Scale-third version and its relationship were examined, with respect to aging (Li 2020). Irrespective of age, the verbal intelligence quotient and verbal comprehension both correlated with striatal DAT binding, but the performance intelligence quotient was not correlated. More specifically, when the striatum was divided into caudate, anterior putamen, and posterior putamen, after incorporating age in the model, only caudate DAT binding remained significantly associated with picture completion subscore in the right caudate, and similarity subscore in the left caudate nucleus. Age related decline of verbal fluency may be known as the ‘tip-of-the-tongue phenomenon’. Other DAT imaging studies in healthy subjects reported that striatal DAT biding and executive function/working memory (Chahine 2016), caudate DAT binding and memory function were correlated (Mozley et al. 2001). Dopamine receptor imaging in healthy subjects showed that D_2_/D_3_ receptor binding was associated with executive function in the caudate (Volkow 1998), and verbal intelligence quotient in the left striatum (Guo 2006). Primate autoradiographic study has demonstrated that the caudate nucleus was the responsible region for the working memory (Levy et al. 1997). These observations from studies of healthy subjects demonstrate that the physiological striatal dopamine function, especially in the caudate nucleus, and perhaps more on the left side, is associated with executive and verbal functions.

Caudate DAT binding of PD patients correlated with phonetic verbal fluency (Polito 2012) and mixed cognitive function such as the trail making test, clock completion and digit span (Nobili 2010). An ^18^F-dopa positron emission tomography (PET) study showed decreased uptake in the anterior cingulate cortex, ventral striatum, and the right caudate nucleus in PD with dementia compared to those without dementia (Ito 2002). ^18^F-dopa PET in PD without dementia showed that right caudate nucleus binding correlated with the Tower of London task, and the left caudate nucleus binding was related to the verbal working memory, which implies a sidedness in the cognitive role of the caudate nucleus (Cheesman 2005). Another ^18^F-dopa PET study in PD demonstrated that caudate binding was correlated with the attention-demanding interference task on the Stroop test (Rinne 2000). Cognitive function related to dopaminergic system includes motor programming, working memory, cognitive flexibility, abstract representation, and temporal analysis/sequencing, all of which serve as executive function (Previc 1999). Imaging using a dopamine D_2_/D_3_ receptor radioligand, ^11^C-FLB457 and PET has shown that the physiological frontal-executive function was mediated by the dopaminergic system, but was disrupted in PD (Ko 2013). ^11^C-raclopride PET, an indirect imaging marker for endogenous dopamine release, was conducted in early stage PD and healthy controls while they performed a working memory and visuomotor control task (Sawamoto 2008). While healthy subjects showed endogenous dopaminergic release in the dorsal caudate while performing a working memory task, this phenomenon was not identified in the early stage PD group, indicating that working memory dysfunction in PD was mediated by the disrupted dopaminergic system in the caudate nucleus. Another ^11^C-raclopride PET study has shown that aerobic exercise in PD increase the endogenous dopamine release in caudate nucleus, which is interesting in view of the mechanism of exercise that its elicit caudate nucleus (Sacheli 2019).

The data driven approach of clinical symptoms and biomarkers in PD has led to dividing PD into mild motor predominant type, diffuse malignant type, and intermediate type. Striatal DAT binding was least affected in the mild motor predominant type and caudate DAT binding was most affected in the diffuse malignant type, which manifests in severe motor symptoms and multiple non-motor symptoms, including cognitive impairment (Fereshtehnejad et al. 2017). Lower caudate DAT binding may predict later decline of executive, visuospatial, and verbal memory function (Arnaldi 2012; Schrag et al. 2017), and freezing of gait (Kim 2018),

Although dopamine replacement improves motor function of PD, several lines of research supports that it also alters cognition. With dopaminergic medications, spatial working memory deficits (which is mediated by right dorsolateral prefrontal cortex) (Cools et al. 2002) and verbal working memory (manipulation phase rather than the retrieval phase of the task) (Lewis et al. 2005), and response inhibition (Wylie 2018) improve in PD. With fMRI, the fronto-striatal region was found to be a responsible region for this improvement (Lewis et al. 2003). Task switching and retrieval process are mediated by caudate nucleus, which projects to the lateral prefrontal cortex, pre-supplementary motor area, and posterior parietal lobe (i.e. mesocortical pathway). Reinforcement learning in an fMRI study in PD has revealed that the dopaminergic replacement diminishes the patient’s emphasis on negative (but not positive) outcomes which improves learning behaviors and was associated with the activation of caudate nucleus (McCoy et al. 2019).

**Fig. 2:**
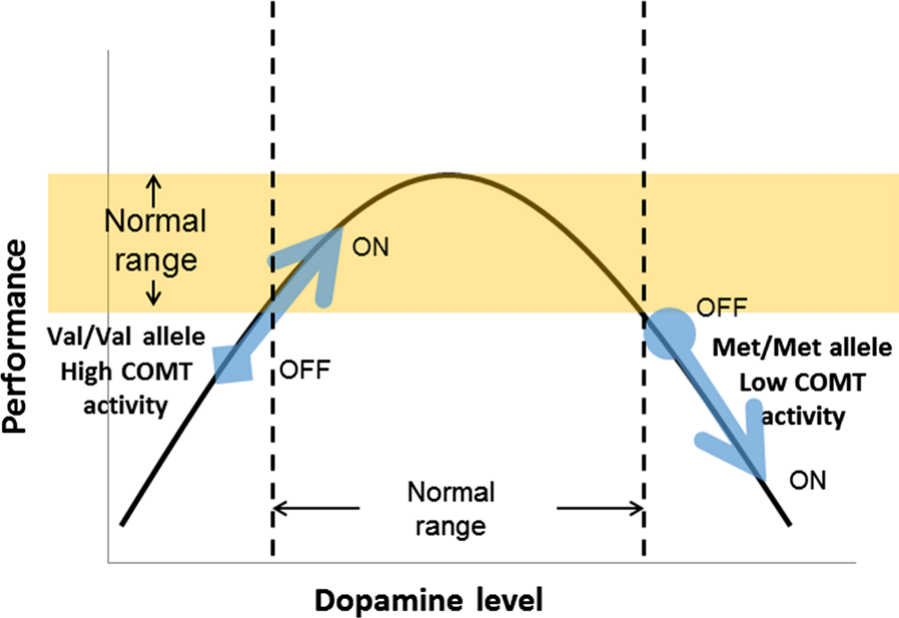
Inverted U-shaped curve relationship between striatal dopamine level and the behavioral, cognitive performance. The effect of dopaminergic treatment (OFF: without medication, ON: with medication) in the cognitive performance differs by the baseline striatal dopamine level and the catechol-O-methyl transferase (COMT) activity, depending on its gene variant

The difference in impaired distributions between these two pathways may lead to a bidirectional response (Cools et al. 2001). Dopaminergic substitution in the affected mesocortical pathway may result in improvement, while relatively unaffected mesolimbic pathway may offer unwanted deleterious effects (Fig. 2). When discussing the dopaminergic function, the Yerkes-Dodson law applies to the relationship between dopaminergic activity and behavioral and cognitive performance. This relationship generally takes the form of non-linear association ‘inverted U-shaped relationship’. If baseline striatal dopaminergic levels are low, dopamine replacement therapy will improve the performance, whereas when baseline striatal dopaminergic levels are high, additional dopamine levels induce poor performance. Optimal performance is obtained at intermediate doses of dopaminergic medication. In this context, working memory performance showed a predictable inverted U-shaped pattern in the prefrontal dopaminergic function via gene variant of *COMT*. *COMT* variants (Met homozygotes have worse cognitive performance which was improved in a longitudinal study (Williams-Gray 2009; Williams-Gray et al. 2007b). Alongside this theory, motor sequence learning deactivates fronto-parietal region in unmedicated PD, which is altered by dopaminergic medication and mediated by *COMT* variant (Argyelan 2008). In summary, caudate dopaminergic system is linked with executive function both in healthy and PD subjects and the dopaminergic effect depends on the baseline striatal dopaminergic levels which is partly controlled by *COMT* variants. However, to my knowledge, cognitive alteration by dopaminergic replacement therapy is not investigated in regard to the striatal dopaminergic status and should be clarified in the future study.

### Ventral striatum

Mediation of rewards, which imply motivation or incentive salience or cost/benefit learning, is dependent on the phasic transmission of dopamine dependent functions in the ventral striatum (nucleus accumbens), The ventral striatum projects to the mesial prefrontal cortex, anterior cingulate cortex (i.e. the mesolimbic pathways) (Bromberg-Martin et al. 2010). The reward learning task is relatively preserved in the unmedicated early stage of PD, but the performance declines with dopaminergic medication, while in the later stage, medication does not worsen the performance (MacDonald 2013), probably due to the remaining baseline dopamine level of ventral striatum. Dopaminergic medication, notably dopamine agonists, when over-dosed, may induce impulse control disorder (pathological gambling, hypersexuality, compulsive shopping, and binge eating) and punding (complex behavior characterized by intense fascination with repetitive movements that traditionally has been determined to be meaningless or purposeless) (Cools 2006; Weintraub et al. 2015). Impulse control disorder is attributable to excessive dopaminergic release in the ventral striatum (Steeves 2009) and subsequent activation of the orbitofrontal cortex (Cilia 2008; Eimeren 2010; Politis 2013). The reward-based decision making is accounted by medial orbitofrontal cortex and punishment-based decision making by lateral orbitofrontal cortex (O'Doherty et al. 2001). Reward prediction are governed by the anterior cingulate cortex, the function of which is preserved in early stage PD, but compromised in advanced PD (Rowe 2008; Kim and Hikosaka 2015). Deep brain stimulation of STN is a device-aided therapy in advanced stage of PD who suffer from motor complications. Although it shows robust efficacy against motor symptoms, cognitive decline and speech problems are recognized as a possible adverse effect. Decline of drawing ability is observed after 3 to 6 months after surgery in half of the PD patients and a perfusional difference before and after surgery was identified in the anterior cingulate cortex, which was not observed in those who did not have altered drawing ability (Furukawa 2020). Thus, postoperative decline of drawing ability may be explained by the aberrant mesolimbic loop by electrical stimulation.

Some of the non-motor symptoms that worsens in the OFF state, such as depression, anxiety, dyspnea, pain, sweating may perhaps be explained by the hypodopaminergic state of mesolimbic dopaminergic system and some symptoms may benefit from the dopamine agonist (Barone 2010; Thobois 2010). In sum, motivation and reward related predictions are explained by aberrant limbic CSPTC loop, in which orbitofrontal and anterior cingulate cortices are involved and is an important viewpoint that clinicians should bear in mind.

## Functional neuroimage and regional consideration of cognitive impairment in PD

### PD cognition-related pattern (PDCP)

By using glucose metabolism PET and spatial covariance analyses, a brain metabolic covariance pattern specific to the cognitive impairment in PD has been identified, the so-called the PD cognition-related pattern (PDCP) (Meles 2015; Schindlbeck and Eidelberg 2018). This PDCP is represented by relative hypometabolism in the frontal and parietal association areas with concurrent relative hypermetabolism in the cerebellar vermis and dentate nuclei. PDCP magnitude is related to executive function (Meles 2015), levodopa-mediated changes in language learning (Mattis et al. 2011), and also caudate dopaminergic activity (Niethammer 2013; Holtbernd 2015). PDCP expression is overexpressed in the MCI stage of PD (Meles 2015), and PD patients with *GBA* gene variants (Schindlbeck 2020). PD subjects also show slight increase in an AD-related abnormal cerebral metabolic pattern, characterized by decreased metabolism in the hippocampus, parahippocampal gyrus, and parietal and temporal association regions. Although the metabolic patterns of PD and AD share some common regions, the two patterns are distinct and specific to the disease (Mattis 2016). PDCP may serve as a biomarker for the diagnosis, monitoring, and early detection of dementia in PD. In the following section, the brain regions included in the PDCP, i.e. frontal cortex, parietal cortex, and cerebellum, are reviewed.

### Frontal cortex

The link between the caudate nucleus and prefrontal cortex has been demonstrated by human resting state fMRI and direct anatomical identification in animal autoradiographic studies (Helmich 2010; Yeterian and Pandya 1991). In PD, an fMRI study has shown that functions in frontal cortex and striatum are abnormally attenuated, which further demonstrated that PD with MCI exhibit under-recruitment in the right caudate nucleus and frontal area (Ekman 2012; Nagano-Saito 2014). Compared to PD without cognitive impairment, those with MCI had lower DAT binding in the right caudate nucleus, which was associated with the brain function of analogous region (Ekman 2012). Alteration in outflow of the caudate nuclei to frontal cortex is related to executive and language function and probably related to the development of MCI in PD.

### Parietal cortex

PD with dementia subjects typically show reduced brain metabolism/perfusion in the posterior parietal region (Firbank et al. 2003; Nobili 2009; Osaki 2009; Baba 2012). Cholinergic imaging with *N*-^11^C-methyl-4-piperidyl acetate (MP4A) and PET is able to quantify the activity of brain acetylcholinesterase, a presynaptic marker of acetylcholine system (Ch4) which originates from the basal forebrain and innervates the entire cortical region. In PD with dementia, ^11^C-MP4A PET illustrated widespread cortical reduction of acetylcholinesterase activity, with propensity to affect the parietal and temporal cortices (Shimada 2009). Notably, cholinesterase PET in AD showed most prominent cholinergic dysfunction in the temporal and parietal regions, similar to the region shown in PD with dementia (Hirano 2018). The cholinergic system has a physiological function of attention, and the efficacy of cholinesterase inhibitors in PD with dementia back up this theory (Yarnall et al. 2011).

Aspects of cognition, which are not influenced by either the dopaminergic replacement or withdrawal in PD, seem to be memory deficits. These dopamine-resistant cognitive functions in PD share common cognitive domains with AD, characterized by memory, language, and constructural impairment and corresponding hypoperfusion in temporal and parietal cortices (Tai 2020).

Heterozygous *GBA* variant carriers of PD, compared to PD without *GBA* variant, showed lower cognitive function, lower ^18^F-dopa PET binding in the caudate nucleus and nucleus accumbens, and in ^18^F-FDG PET, higher PDCP expression and lower glucose metabolism/perfusion in bilateral medial and lateral parietal lobes (Cilia 2016; Greuel 2020). Heterozygous *GBA* variant carriers of PD is associated with Lewy body pathology, commonly compromise executive and visuospatial but not memory function, which segregates from AD by both clinical and pathological aspects, although it is intriguing that its cortical dysfunction was observed in the bilateral medial and lateral parietal lobes (Tsuang 2012; Mata 2016). This may not fit into the dual syndrome hypothesis, which may imply the heterogeneity of the pathophysiology of dementia in PD. This observation should further be clarified by evaluating the earlier stage *of GBA* variant PD carrier.

Collectively, evidence from functional imaging, cholinergic imaging, and *GBA* variant, has led to the notion that parietal and temporal regions in PD are the key region for the development of dementia in PD, in some cases with concurrent AD pathology.

### Cerebellum

Although increased activity of cerebellum in PD can appear to be present as a result by the global normalization approach, this section will discuss the cognitive aspects of cerebellum in PD. Cerebellar overactivity relating to the motor symptoms in PD (as well as dystonia) has been repeatedly observed and has thought to be involved in the motor control (Quartarone 2020). The cerebellum is responsible for sequencing detection (Leggio and Molinari 2015). It detects the accordance/discrepancy between the predicted and actual stimulus (Molinari et al. 2018). If the incoming stimulus corresponds to the predicted one, the cerebellar outputs is minimal, whereas if it was discrepant, cerebellar outputs are activated. Cerebellar activation in healthy subjects involves working memory, language and executive function (Molinari et al. 2018; Marvel and Desmond 2010; Mariën et al. 2018), all common cognitive domains involved in PD. Cerebellar damage may also lead to an impairment of attentional control, emotional control, social skill set, in addition to psychotic symptoms, the so-called ‘cerebellar cognitive affective syndrome’ (Schmahmann and Sherman 1998; Schmahmann et al. 2007). Interestingly, a placebo effect in PD has been visualized by network analysis, showing that activity in the cerebellar vermis, and subgenual anterior cingulate cortex increased after sham-surgery, specifically related to those who benefit from the procedure (Ko 2014). Similarly, reward magnitude was correlated with cerebellar vermis activation in PD, while prefrontal, rhinal cortices and thalamus were activated in the healthy control, demonstrated by perfusion PET (Goerendt et al. 2004).

Although the cerebellum is not included in the Braak’s hypothesis, recent finding demonstrated Lewy pathology, predominantly Lewy neurites and to a lesser degree, Lewy bodies, in the cerebellar structures, such as central nucleus and deep white matter of cerebellum in PD and DLB (Seidel 2017). In this respect, cerebellum in PD may play certain role in the cognitive and/or mental function, which remains to be clarified in the future studies.

## Dual syndrome hypothesis

Intellectual function in healthy subjects is related with fronto-parietal cortices, in that as higher intelligence showed shorter path length in resting state fMRI (Heuvel et al. 2009). In healthy subjects, the frontal and parietal cortex have robust connectivity with the internal segment of globus pallidum, an output region for the basal ganglia (Cacciola 2019). Functional connectivity, evaluated by resting state fMRI, demonstrated that reduced connectivity in PD was found in the frontal region, whereas in dementia with Lewy bodies group in the parieto-occipital region (Borroni 2015). Interestingly, PD medication OFF state was associated with compensatory mechanisms, such as cognitive reserve, in the frontal cortex (Shine 2019).

**Fig. 3:**
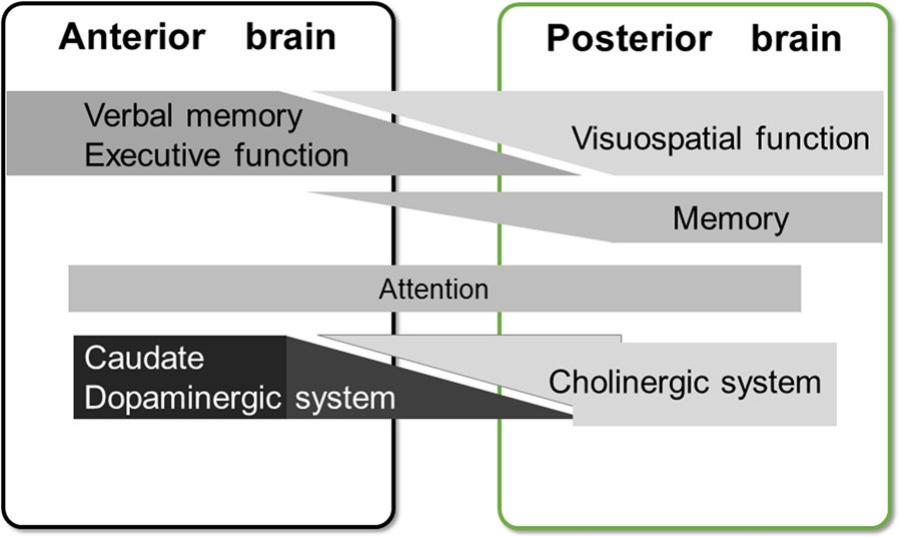
Schematic diagram of dual syndrome hypothesis (modified from Kehagia et al. (2013))

Kehagia and coworkers (Kehagia et al. 2013) have introduced an idea of ‘dual syndrome hypothesis’ for explaining the integrity of symptoms and underlying pathophysiology of cognitive impairment in PD. Accumulated evidence supports the hypothesis that dichotomized brain system are independent but partially overlap. Verbal memory and executive dysfunction relate to changes in the anterior brain system and are usually related to MCI in PD, whereas the visuospatial and memory dysfunction in the posterior brain system relate to dementia of PD (Fig. 3). Anterior brain system refers to the fronto-striatal dopaminergic system via the caudate nucleus, and the posterior brain system refers to the temporal, parietal, occipital cortices and cholinergic system. Impairment of anterior brain system may also relate to tremor dominant phenotype while the posterior brain system may relate to postural instability and gait disturbance phenotype. By understanding this concept, one might more efficaciously manage the heterogeneous cognitive symptoms in PD.

## Conclusions

The cognitive heterogeneity of PD may partly be explained by dual syndrome hypothesis. Understanding fronto-striatal dopaminergic system and posterior brain cholinergic system may lead to more targeted treatments. However, since most treatment strategies in PD put weight on the motor improvement, it may be difficult to optimize both cognitive and motor functions by the systemic action of a given dose of dopaminergic medication. Local modification is required to overcome this problem and therapies such as deep brain stimulation, gene-target therapy, or non-dopaminergic medication may be needed to regulate functions related to cognition in PD.

## Data Availability

None.

## Abbreviations

AD: Alzheimer’s disease
COMT: Catechol-O-methyl transferase
CSPTC: Cortico-striatal-pallido-thalamo-cortical
DAT: Dopamine transporter
fMRI: Functional magnetic resonance image
GBA: Glucocerebrosidase
MCI: Mild cognitive impairment
PD: Parkinson’s disease
PDCP: Parkinson’s disease cognition-related pattern
PET: Positron emission tomography
STN: Subthalamic nucleus
SPECT: Single photon emission computed tomography
